# Incidence of major structural cardiac defects in fetuses with increased nuchal translucency and normal karyotype

**DOI:** 10.1186/1749-8090-8-S1-P83

**Published:** 2013-09-11

**Authors:** K Praprotnik, BM Gersak

**Affiliations:** 1 Diagnostic Centre Strah, Domzale, Slovenia

## Background

Nuchal translucency (NT) is the sonographic appearance of a collection of fluid under the skin behind the fetal neck in the first-trimester of pregnancy. In fetuses with chromosomal abnormalities, cardiac defects and many genetic syndromes NT thickness is increased. The aim of the study was to evaluate the incidence of major cardiac defects in relation to the fetal NT in chromosomally normal fetuses in an unselected Slovenian pregnant population.

## Methods

During a 5-year period (January 4, 2005 to April 30, 2010) 11,980 pregnant women were appointed for the first trimester ultrasound screening examination at a single outpatient clinic. The women with an increased risk for chromosomal anomalies (≥ 1:300) calculated on the basis of maternal age, NT and fetal crown-rump length were offered invasive testing for fetal karyotyping. The fetuses with increased fetal NT and normal karyotype were followed by detailed structural ultrasound evaluation between the 20th and the 24th week of gestation.

## Results

Five hundred and fifty-eight fetuses had an increased fetal NT and normal karyotype (558/11,980; 4.7%). In 46 cases (46/558; 8.2%) the outcome of the pregnancy was unknown; therefore 512 singleton pregnancies were included in the further analysis. Among them we found 50 fetuses (9.4%; 48/512) with either single or multiple abnormalities. There were 10 cases (20.8%, 10/48) with heart defects (1 case of atrioventricular septal defect, 1 case of coarctation of the aorta with VSD, 3 cases of ventricular septal defect, 2 cases of hypoplastic left heart syndrome, 2 cases of isolated valve anomaly, one case of Fallot’s tetralogy). In 2 cases pregnancy was terminated at parental request (Fallot’s tetralogy, hypoplastic left heart syndrome).

## Conclusion

Our data confirm that increased NT during the first trimester is associated with major cardiac defects in fetuses with a normal karyotype and the risk of a cardiac defect increases markedly with increasing NT.

